# Prospective cohort study on mesh shrinkage measured with MRI after robot-assisted minimal invasive retrorectus ventral hernia repair using an iron-oxide-loaded polyvinylidene fluoride mesh

**DOI:** 10.1007/s00464-023-09938-3

**Published:** 2023-02-28

**Authors:** Maaike Vierstraete, Roel Beckers, Lorenz Vangeel, Brend Foriers, Pieter Pletinckx, Filip Muysoms

**Affiliations:** 1 Department of Surgery, Maria Middelares Hospital, Buitenring Sint-Denijs 30, 9000 Ghent, Belgium; 2 Department of Radiology, Maria Middelares Hospital, Buitenring Sint-Denijs 30, 9000 Ghent, Belgium

**Keywords:** Ventral hernia, Retrorectus, Retromuscular, Visible mesh, Polyvinylidene fluoride, Mesh shrinkage

## Abstract

**Background:**

Mesh-reinforced ventral hernia repair is considered the gold standard treatment for all but the smallest of hernias. Human data on mesh shrinkage in the retrorectus mesh position is lacking. A prospective observational cohort study was performed to measure mesh shrinkage in robot-assisted minimal invasive retrorectus repair of ventral hernias.

**Methods:**

A cohort of 20 patients underwent a robot-assisted minimal invasive retrorectus repair of their ventral hernia. Magnetic resonance imaging (MRI) imaging was performed one month and thirteen months after implantation of an iron-oxide-impregnated polyvinylidene fluoride (PVDF) mesh to assess the decrease in mesh surface area. Inter-rater reliability among three radiologists regarding measurement of the mesh dimensions was analyzed. Quality of Life scoring was evaluated.

**Results:**

The inter-rater reliability between the radiologists reported as the intra-class correlations proved to be excellent for mesh width (ICC 0.95), length (ICC 0.98) and surface area (ICC 0.99). Between MRI measurements at one month and thirteen months postoperatively, there was a significant increase in mesh surface area (+ 12.0 cm^2^, *p* = 0.0013) and mesh width (+ 0.8 cm, *p* < 0.001), while the length of the mesh remained unchanged (−0.1 cm, *p* = 0.754). Quality of Life Scoring showed a significant improvement in Quality of Life after one month and a further improvement at thirteen months (*p* < 0.001).

**Conclusion:**

There was an excellent inter-rater reliability between three radiologists when measuring width, length, and surface area of an iron-oxide-impregnated PVDF mesh using MRI visualization. Mesh shrinkage was not observed, instead the effective mesh surface area and width of the mesh increased.

**Supplementary Information:**

The online version contains supplementary material available at 10.1007/s00464-023-09938-3.

## Background

Mesh-reinforced ventral hernia repair reduces the rates of hernia recurrence and is considered the gold standard treatment for all but the smallest of ventral hernias [1, 2]. Multiple mesh products are currently available and mesh shrinkage is believed to play a potential role in the incidence of hernia recurrence. Data on mesh shrinkage are mainly derived from animal studies with highly variable rates of shrinkage ranging from 10.2 to 41.0% [3, 4]. The diversity of animal models, mesh properties, anchoring devices and study observation times has led to inconsistent results, making its translation to human practice questionable. Human studies are scarce and the majority report on mesh shrinkage in the intraperitoneal mesh position with a reported decrease in transverse mesh diameter of 0.1–10.6% [5–9]. However, these results cannot automatically be used to estimate the shrinkage rate of meshes in the extraperitoneal or retrorectus position as the location of the implanted mesh is believed to play an important role in mesh behavior. Two previous studies where mesh shrinkage was analyzed in open ventral hernia repair using a retrorectus mesh position illustrated that the overall mean mesh surface area increased by about 10% [10, 11]. The new method of incorporating iron-oxide particles into meshes allows for precise mesh depiction on postoperative magnetic resonance imaging (MRI) and has been previously studied in studies with intraperitoneal mesh placement [8, 12].

## Objectives

This study aims to assess the change in mesh surface area, between 1 and 13 months after implantation of an iron-oxide-impregnated polyvinylidene fluoride (PVDF) mesh (DynaMesh®-CICAT visible, FEG Textiltechnik, Aachen, Germany) in robot-assisted minimal invasive retrorectus ventral hernia repair by means of MRI visualization. Inter-rater reliability among radiologists regarding measurement of mesh dimensions via MRI will be assessed.

## Materials and methods

### Study design

The study is a prospective single-center observational cohort study on mesh shrinkage measured with MRI after robot-assisted minimal invasive retrorectus hernia repair. This manuscript is written in accordance with the STROBE statement (Strengthening the Reporting of Observational Studies in Epidemiology) [13].

### Setting

The study was performed at the Department of Surgery of Maria Middelares Hospital in Ghent, Belgium. The study was approved prior to the first enrolment by the ethical medical review board of the Antwerp University Hospital with the trial number B300201733600 and was registered at ClinicalTrials.gov (NCT03380312) on December 2017 with the acronym IRMA (MRI imaging of ipsilateral retrorectus access) study. Patients were included between 9 January 2018 and 19 June 2020. A sample size of 20 patients was considered sufficiently large to allow for an accurate mesh shrinkage measurement and small enough to be performed within a reasonable timeframe and the available budget. Informed consent was signed prior to the surgical procedure. All patients were operated by a single surgeon experienced in both laparoscopic and robot-assisted ventral hernia repair (FM). If no major comorbidities were present, operations were planned as outpatient procedures. MRI examinations at 1 month and 13 months post-operatively were carried out by one radiologist committed to abdominal wall imaging (RB).

### Participants

The study sample consisted of patients aged more than 18 years presenting with a midline ventral hernia, location M2-M3-M4 according to the European Hernia Society classification [14]. Exclusion criteria were lateral (L1-L4) hernias; subxiphoid (M1) hernias; suprapubic hernias (M5); large midline hernias requiring additional component separation; emergency surgery; ASA score > 4; clean-contaminated or contaminated procedures, concomitant procedures, life expectancy of less than 2 years, contra-indications for MRI and previous mesh-reinforced ventral hernia repair.

### Surgical technique

All operations were performed under general anesthesia. Each procedure was performed in exactly the same manner applying the previously described robot-assisted transabdominal retrorectus umbilicial prosthetic repair (rTARUP) technique [15]. DynaMesh®-CICAT visible (FEG Textiltechnik, Aachen, Germany) mesh was used in all patients. DynaMesh®-CICAT visible mesh consists of monofilament PVDF enhanced with paramagnetic iron-particles to provide for MRI visibility. It is designed for abdominal wall and umbilical hernia repair with an extraperitoneal mesh position and is classified as a large pore monofilament mesh, class Ia according to Klinge’s classification [16].

### Imaging technique and data measurement

The MRI examination was performed with patients in prone position and feet first orientation using a 3 T magnetic resonance scanner (Philips Achieva, Best Netherlands). In this way the abdominal wall is closest to the posterior coil integrated in the table and abdominal wall motion is reduced. The study protocol takes about 11–12 min and consists of scout view, Coronal T1 TFE, Sagittal IP FFE, Coronal 3DT1, Coronal T2 TSE and Axial T2 TSE. Only the last sequence is performed in two breath holds, the other sequences are performed during free breathing. Coronal T1 TFE is used for accurate planning of the sagittal IP FFE. Both sequences are used to plan the 3DT1 sequence. Both sagittal IP FFE and coronal 3DT1 were used to create thick MINIP (minimal intensity projection) images of the total mesh surface.

**Fig. 1:**
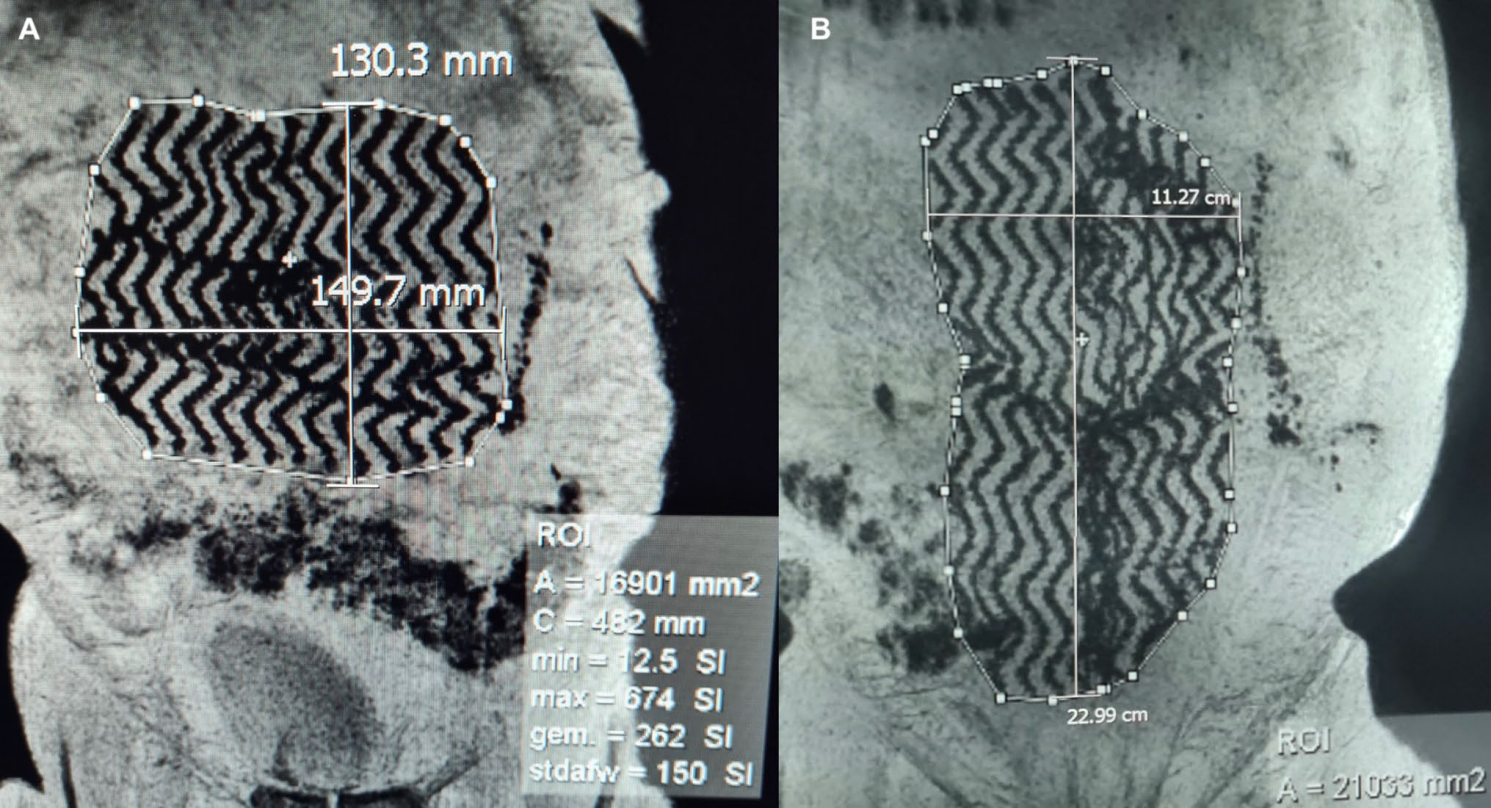
3DT1 MRI imaging in robot-assisted minimal invasive retrorectus hernia repair using an iron-oxide-loaded PVDF mesh. Calculations of the mesh surface area and maximal orthogonal diameters (width and length) are performed by drawing mesh contours following the endpoint of the mesh wires. Note the remarkable streak of iron-loaded particles at the left lateral border of the mesh

Drawing mesh contours following the endpoint of the mesh wires resulted in calculation of the projected surface and circumference of the mesh. Additionally maximal orthogonal diameters were measured (Fig. 1). The correct localization of the mesh was evaluated as well as the presence of surgery-related complications as seroma, hematoma, or recurrences.

### Bias

All images were pseudonymized and blinded to the patients’ identity and MRI timing. Each radiologist received a randomly ordered scan series to perform the measurements and thus was blinded to the timing of the MRI scan, either at 1 or 13 months postoperatively. Also, the scans of the same patient were not reviewed subsequently to avoid bias.

### Outcome variables

The primary outcome of this study was the change of the mesh surface area, as measured with MRI between 1 and 13 months after implantation of the retrorectus iron-oxide-impregnated PVDF mesh. Secondary outcome measures were the change in mesh width and length between 1 and 13 months postoperatively as well as the inter-rater reliability among the three radiologists regarding mesh measurement with MRI.

Standard procedures for all perioperative care from patient, surgical and anaesthetic perspectives were followed. Any deviations from the normal postoperative course (intra-operative, early post-operative and late postoperative course) were analyzed and classified according to the Clavien-Dindo classification of surgical complications. All patient data were entered in the prospective European Registry of Abdominal Wall Hernias (EuraHS) online database [17].

Patients were examined at 1 and 13 months by the same surgeon (FM) and were asked to report any complaints (such as lasting pain at rest and/or during activity, discomfort, or other irregularities since the last contact). A clinical assessment with the Valsalva maneuver was performed. Patients were asked to complete the EuraHS Quality of Life questionnaire (QoL) at each clinical visit.

### Statistical analysis

The statistical analysis was performed by an independent statistician. Regarding descriptive statistics, categorical variables were reported in terms of frequency (*n* (%)), while continuous variables were presented as median with interquartile range (IQR) for non-Gaussian variables or mean ± the standard deviation (SD) for Gaussian variables. The inter-rater reliability between the three radiologists was evaluated by calculating intra-class correlations (ICC with 95% confidence interval) for the surface area (cm^2^), width (cm) and length (cm) in the pooled sample of the 40 MRI investigations. Inter-rater reliability is considered excellent if the ICC exceeds the value 0.90. Change in mesh size between 1 and 13 months was reported as the mean value ± SD and its statistical significance was evaluated according to the paired T-Test. Spearman rank correlation coefficients were calculated to explore patients’ characteristics in their relation to mesh shrinkage. Changes in EuraHS QoL scores, preoperatively and at 1 and 13 months postoperatively, were evaluated using linear mixed modeling using an unstructured covariance structure. A type I error level of α = 0.05 was used to indicate statistical significance. All statistical analyses were performed using SAS statistical software (release 9.4).

## Results

### Participants

Twenty-two eligible patients were screened between 9 January 2018 and 19 June 2020. One patient declined participation and one patient was excluded because of contra-indication for MRI scanning (cerebral aneurysm clipping). One patient did not attend the clinical follow-up visit at 13 months but did have the MRI follow-up performed correctly. A study flow diagram is depicted in Supplementary material Fig. 1.

### Descriptive data

Patients’ demographics, intra-, pre- and postoperative data are shown in Table 1. The majority of patients were male and half of them were obese (body mass index (BMI) ≥ 30 kg/m^2^). All hernias were either primary or incisional hernias located in zones M2-M3-M4. Only one study patient had a recurrent ventral hernia. The hernia defect was closed in all patients with a slowly absorbable barbed suture (V-Loc™ 2.0 (Covidien, North Haven, CT)). In 18 out of the 20 (90%) patients the mesh was fixed with an absorbable suture, either Vicryl® 3.0 (Ethicon Products, Amersfoort, the Netherlands) or V-Loc™ 3.0 (Covidien, North Haven, CT). In 14/20 (70%) patients a mesh of 15 cm by 15 cm was used. In the other cases, the mesh was tailored in a rectangular fashion to maintain sufficient hernia defect overlap.

**Table 1:** Patient characteristics, intra-operative data, and postoperative clinical outcome in a prospective cohort study on robot-assisted minimal invasive retrorectus hernia repair using an iron-oxide-loaded PVDF mesh in 20 patients

	*n*/*N* (%) or mean (SD)
Patient demographics	
Age (years)	57 (10.7)
Male	16/20 (80)
BMI (kg/m^2^)	30 (4.9)
< 25 kg/m^2^	6/20 (30)
25–29.9 kg/m^2^	4/20 (20)
≥ 30 kg/m^2^	10/20 (50)
Patient variables	
Smoker	2/20 (10)
Diabetes	2/20 (10)
Pulmonary disease	3/20 (15)
Renal disease	1/20 (5)
Hernia variables	
Hernia classification	
Primary epigastric	1/20 (5)
Primary umbilical	11/20 (55)
Incisional hernia (M2-M3-M4)	8/20 (40)
Width of the hernia (cm)	2.7 (0.86)
Length of the hernia (cm)	2.4 (1.04)
Intra-operative data	
Duration of the surgery (min)	82 (20)
Width of the mesh (cm)	15.9 (2.0)
Length of the mesh (cm)	16.9 (4.0)
Mesh fixation	18/20 (90)
Postoperative data	
Postoperative complication	
Prolonged hospital stay for pain control medication	1/20 (5)
Hospital stay (days)	
0	15/20 (75)
1	4/20 (20)
2	1/20 (5)
Complications at 1 month follow-up	3/20 (15)
Seroma	3/20 (15)
Complications at 13 months follow-up	0/20 (0)
Recurrence at 13 months follow-up	0/20 (0)

### Outcome data

**Fig. 2:**
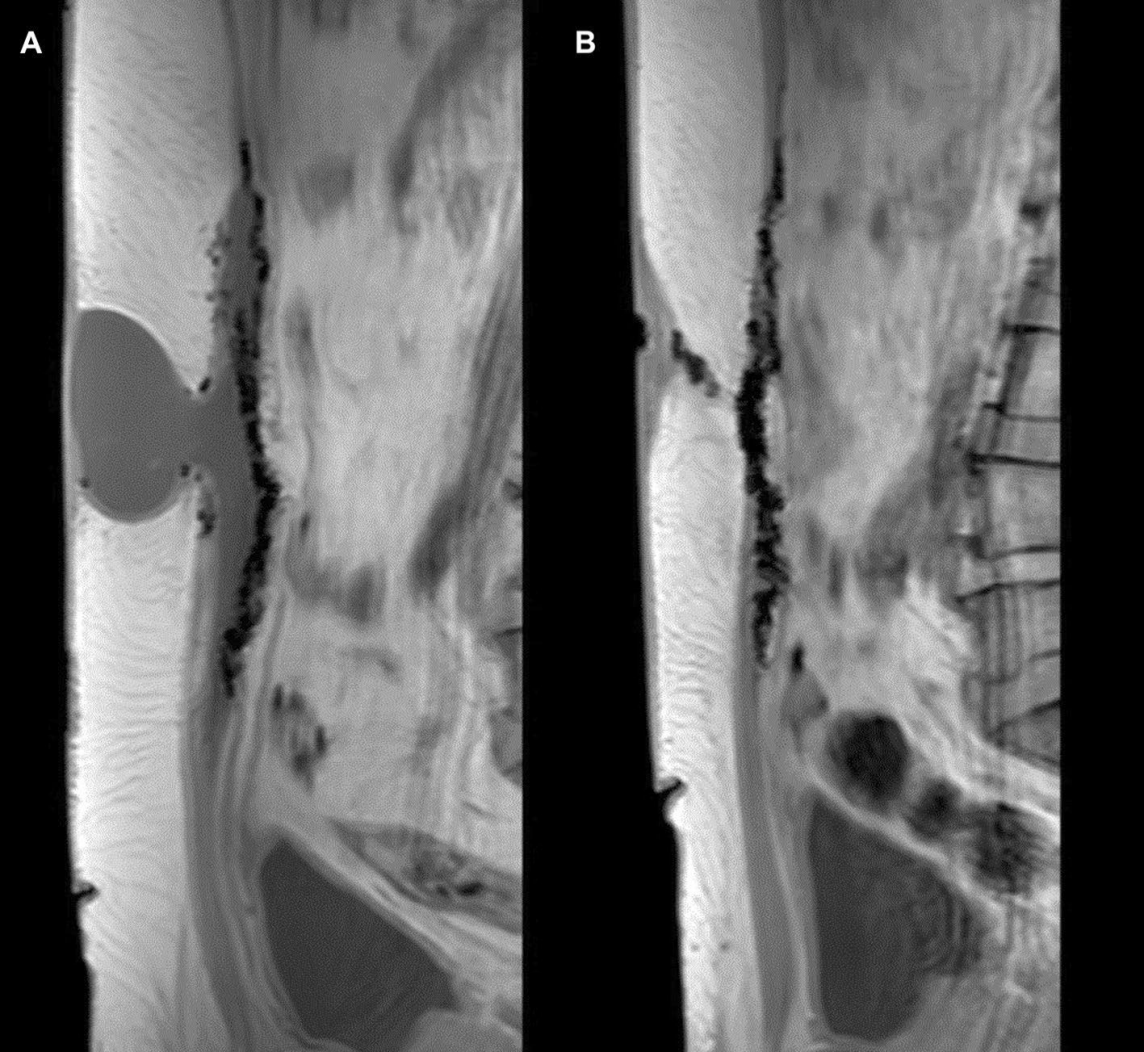
3DT1 MRI imaging in a patient who had a robot-assisted minimal invasive retrorectus ventral hernia repair using an iron-oxide-loaded PVDF mesh **A** MRI image at 1 month showing a postoperative seroma and a dehiscence of the hernia defect closure after an episode of heavy coughing **B** No residual seroma on MRI at 13 months postoperatively

Fifteen patients were operated in an outpatient setting. One patient stayed for two nights because of persisting pain (Grade I according to the Clavien-Dindo Classification). At 1 month follow-up, three patients had a clinically present seroma which was followed without requiring any intervention. One patient mentioned an episode of heavy coughing with a sensation of something snapping at the hernia site. MRI examination did confirm dehiscence of the hernia defect closure and the presence of a seroma (Fig. 2). No residual seromas were noted at 13 months. QoL scoring using the EuraHS QoL questionnaire showed a significant improvement in overall QoL as well as for all three domains (pain, restrictions of activities, and cosmetic discomfort) after 1 month compared with preop values. In addition, a further improvement was noted at 13 months follow-up. Detailed data on the QoL results are available as supplementary material (Supplementary Table 1 and Supplementary Fig. 2).

### Main results

The measurements of the primary endpoint are shown in Table 2. MRI measurements at 1 month and 13 months postoperatively showed a significant increase in mesh surface area (+ 12.0 cm^2^, *p* = 0.0013) and mesh width (+ 0.8 cm, *p* < 0.001), while the length of the mesh remained unchanged (−0.1 cm, *p* = 0.754).

**Table 2:** 3DT1 MRI mesh size calculations per radiologist on 1 month and 13 months postoperatively in a prospective cohort study on robot-assisted minimal invasive retrorectus hernia repair with an iron-oxide-loaded PVDF mesh

	At 1 month Mean (SD)	At 13 months Mean (SD)	Change Mean (SD), *p*-value
*3DT1 Width (cm)*	*15.3 (2.0)*	*16.1 (1.6)*	+ *0.8 (0.7), p* < *0.001*
Radiologist 1	15.2 (2.0)	16.1 (1.5)	+ 0.7 (0.9), *p* = 0.0016
Radiologist 2	15.4 (2.1)	16.2 (1.6)	+ 0.8 (1.0), *p* = 0.0016
Radiologist 3	15.3 (1.9)	16.0 (1.8)	+ 0.7 (0.8), *p* < 0.001
*3DT1 Length (cm)*	*15.0 (3.6)*	*14.9 (3.1)*	−*0.1 (0.9), p* = *0.754*
Radiologist 1	14.9 (3.7)	14.8 (3.1)	−0.0 (0.8), *p* = 0.868
Radiologist 2	15.2 (3.8)	15.0 (3.0)	−0.2 (1.4), *p* = 0.468
Radiologist 3	14.8 (3.6)	14.9 (3.2)	+ 0.1 (0.7), *p* = 0.694
*3DT1 Surface area (cm*^*2*^*)*	*199.2 (65.0)*	*211.3 (57.0)*	+ *12.0 (14.2), p* = *0.0013*
Radiologist 1	198.0 (65.9)	210.0 (56.3)	+ 12.1 (14.8), *p* = 0.0018
Radiologist 2	200.9 (64.8)	213.1 (56.3)	+ 12.2 (14.8), *p* = 0.0016
Radiologist 3	198.0 (64.5)	210.7 (58.6)	+ 11.8 (14.3), *p* = 0.0016

The overall data from all 3 radiologists are reported in italics

Based on all 40 3DT1 MRI investigations, inter-rater reliability between the three radiologists reported as the intra-class correlations (ICC (95% CI)) proved to be excellent for mesh width (0.95 (0.92–0.97)), length (0.98 (0.97–0.99)) and surface area (0.99 (0.99–1.00)). However, measurement of the length (*p* = 0.0035) and consequently surface area (*p* < 0.001) was significantly larger for radiologist 2 in comparison to the other radiologists (Supplementary Table 2).

### Other analyses

A significant difference in mean (SD) mesh surface area of 2.6 (0.5) cm^2^ between the IP FFE and 3DT1 sequences was found (*p* < 0.001) (202.6 cm^2^ IP FFE versus 205.2 cm^2^ 3DT1). This finding was consistent for all three radiologists. 3DT1 MRI imaging was considered the most valid from a radiological point of view.

**Fig. 3:**
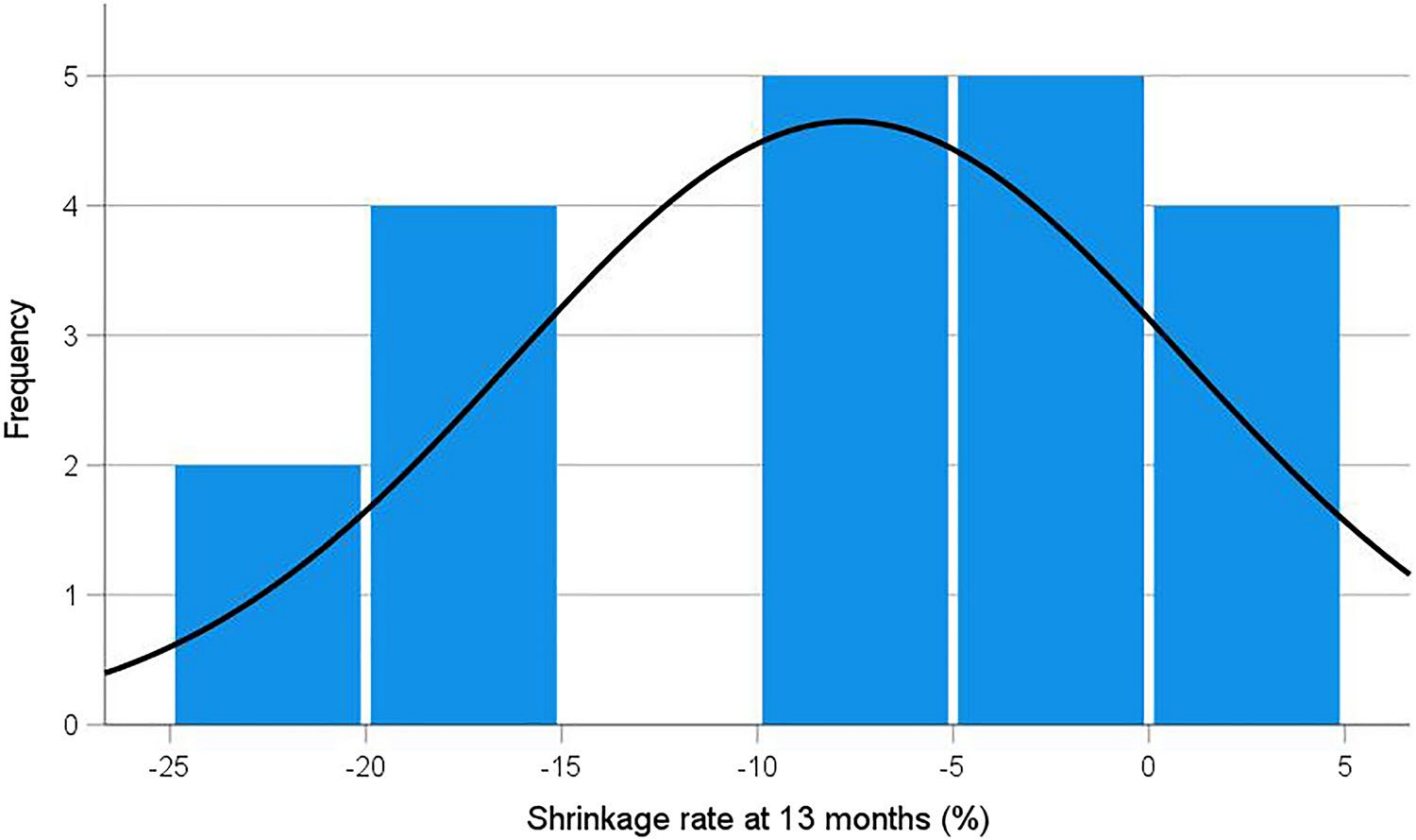
Histogram on the shrinkage rates between 1 and 13 months after robot-assisted minimal invasive retrorectus hernia repair using an iron-oxide-loaded PVDF mesh, in relation to the number of patients

Figure 3 depicts a histogram of the mesh shrinkage rates between 1 and 13 months, calculated as proposed in our previous study.^8^ In six patients the mesh surface area increased by more than 15% (negative shrinkage rate) against a decrease of mesh surface area of less than 5% in four patients (positive shrinkage rate).

**Fig. 4:**
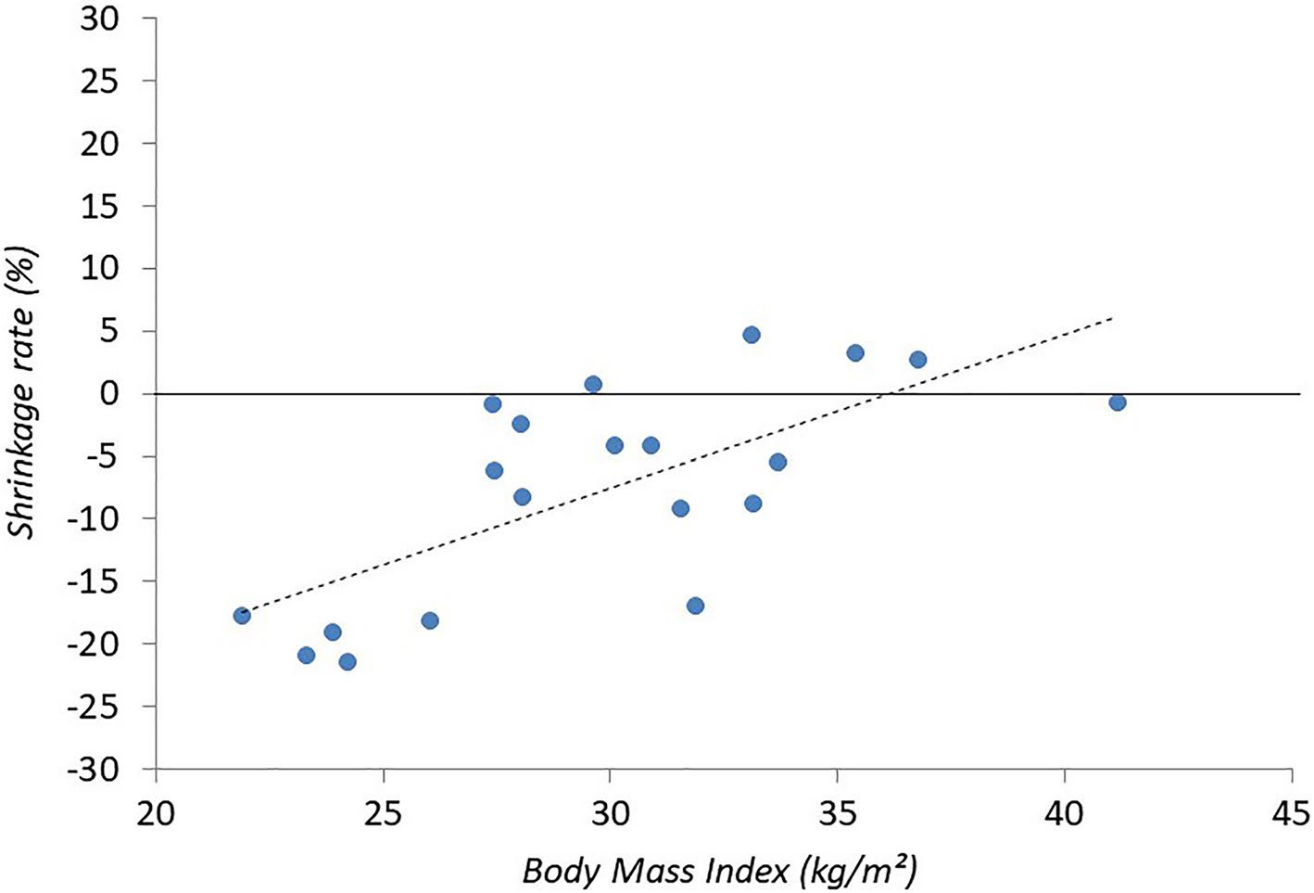
Graphical illustration on the degree of mesh shrinkage after robot-assisted minimal invasive retrorectus ventral hernia repair in relation to the patients’ body mass indices at the time of the surgery

The mesh shrinkage rates showed a positive association with the initial width of the implanted mesh (Spearman ρ = 0.65), the width of the mesh at 1 month (Spearman ρ = 0.65), the length of the mesh at 1 month (Spearman ρ = 0.56) and the body mass index (Spearman ρ = 0.67). The dimensions of the hernia itself, were not significantly associated with the shrinkage rate. As illustrated in Fig. 4, the changes in mesh surface area were more pronounced in patients with a lower BMI at the time of the surgery.

## Discussion

### Key results

We found no significant shrinkage of PVDF mesh in the retrorectus position measured on MRI at 1 and 13 months postoperatively. We even found a negative shrinkage rate (increase) of the mesh surface area and the width of the mesh. This negative shrinkage rate was correlated with a lower BMI.

### Limitations

Our study had a relatively limited sample size of 20 patients, which is comparable with other human studies on mesh shrinkage. Although a significant increase in mesh surface area and mesh width was demonstrated, one might assume that in case of a larger sample size, a statistically significant difference in mesh length might be seen over time as well. However, the clinical relevance of the latter is questionable as we assume that mesh shrinkage should be determined on measurements of the total mesh surface area. The current findings are only applicable for ventral midline hernias, location M2-M3-M4 according to the European Hernia Society (EHS) classification [14]. These results cannot be extrapolated for lateral, subxiphoid, or suprapubic hernias because of their higher degree of mesh bending, besides different dynamic movements of the abdominal wall in lateral hernias which might influence mesh behavior.

### Interpretation

In ventral and incisional hernia repair, the potential for shrinkage of meshes after implantation remains a concern. Data from experimental studies on animals and, to a lesser extent, humans, is inconsistent due to the diversity of the different models, mesh properties, anchoring devices, study times, assessment of the mesh sizes, and different definitions for mesh shrinkage, in addition to a high level of bias due to the retrospective design of the majority of the studies [3–9]. The clinical relevance of mesh shrinkage remains unclear; however, it is considered important to assure adequate overlap of the mesh with respect to the size of the hernia defect to compensate for possible contraction of the mesh. After implantation, the unavoidable host’s cellular response to the mesh warrants a complex inflammatory process responsible for tissue incorporation and scar formation which might account for a substantial shrinkage of the mesh area [16].

Most studies in humans note mesh shrinkage in the intraperitoneal mesh position where the mesh is fixed with transfascial sutures and/or permanent or resorbable tackers [5–9]. Besides the well-known concern of bowel adhesions with intraperitoneal mesh placement, mesh fixation with transabdominal sutures can cause severe pain likely due to nerve entrapment [18–20]. In our study group, 18/20 (90%) of the meshes were fixed with absorbable sutures; however, the need for mesh fixation should be determined individually by the surgeon. The retrorectus plane is believed to allow for a flat mesh placement while creating a good environment for mesh incorporation which is further facilitated by the intraabdominal pressure.

Most patients can be treated in an outpatient setting. In our relatively small patient group, one patient had a prolonged hospital stay due to pain, while three patients had a postoperative seroma where conservative treatment was sufficient. One of the latter had a suture rupture at the site of the hernia defect closure due to heavy coughing. None of them presented with postoperative small bowel obstructions or mesh-related complications during the 13 months of follow-up. There was a significant improvement in QoL score at 1 month compared to preoperative values. In addition, a further improvement was noted at 13 months follow-up. Our short-term results confirm the finding that minimally invasive retrorectus ventral hernia repair has consolidated its position over the last decade as a safe and effective technique [21].

Two previous studies reported outcomes on mesh shrinkage in retrorectus ventral hernia repair via an open approach. Langer et al. analyzed mesh shrinkage in 50 patients who had an open retromuscular mesh repair with either a heavyweight (Biomesh P®) or lightweight (NK®) polypropylene mesh loaded with barium sulfate. During a follow-up period of two-years, X-ray and CT imaging identified mesh shrinkage in 4/50 (8%) patients (3, 8, 8 and 22.2%, respectively). In all other patients 46/50 (92%), a significant increase in the mesh surface area and transverse diameter was seen over time (*p* < 0.0001 and *p* < 0.001, respectively). Results were the same for heavyweight and lightweight meshes (*p* = 0.121) [11]. Similarly, Rogmark et al. reported their data on 17 patients who had an open retrorectus ventral hernia repair (ProLite™, Atrium). By performing an abdominal X-ray in the supine position within 2 days postoperatively and after 1-year of follow-up, they recorded an increase in mesh area of 0.5% along with an increase of 3.1% in the transverse mesh distance and a decrease in longitudinal mesh distance of 2.6% [10]. Likewise, in our patient group of 20 patients who had a robot-assisted minimal invasive retrorectus ventral hernia repair, MRI calculations between 1 and 13 months postoperatively, showed a significant increase in mesh surface area (+ 12.0 cm^2^, *p* = 0.0013) and transverse width (+ 0.8 cm, *p* < 0.001) while the longitudinal distance remained unchanged (−0.1 cm, *p* = 0.754). Only 4/20 (20%) patients had a mesh shrinkage of less than 5%, whereas all other patients had an increase of their mesh surface area (25% at maximum). This unexpected finding, in line with the two other human studies, suggests that tissue incorporation of the mesh in a retrorectus position is not associated with a considerable rate of mesh shrinkage. Contraction of the mesh alone is thus unlikely to cause recurrence if an adequate mesh placement is performed in the retrorectus position. Since most of the recurrences after incisional hernia repair are located around the border of the mesh, technical factors such as dislocation, inadequate overlap of the mesh, or missed defects are of paramount importance [22].

A positive association between the change in mesh surface area and BMI (Spearman ρ = 0.67) was found, illustrating that changes in surface area were more pronounced in patients with a lower BMI at the time of the surgery (Fig. 4). No record of changes in BMI were recorded during follow-up; however, extension of the mesh might be attributed to major weight gain or repetitive expansion of the abdominal wall (e.g., breathing, coughing, lifting weights, etc.). It is believed that frequent or sustained distention of the abdominal wall will elongate the tissues with embedded meshes; the elasticity of the mesh will be overcome and result in a plastic permanent deformation of the mesh [23]. Besides, proper orientation of anisotropic meshes (e.g., DynaMesh®-CICAT visible with its green orientation strips along the longitudinal direction) within the anisotropic abdominal wall is a prerequisite to improve the mechanical compatibility between the mesh and the physiological movement of the abdominal wall [23, 24]. The exact clinical implication of these findings is yet to be established.

Although the sample size of our study is not very large, our prospective study demonstrates that mesh contraction in retrorectus robot-assisted minimal invasive ventral hernia repair with an iron-oxide-impregnated PVDF mesh, was of no significant relevance. These findings are in line with the results of Rogmark et al. and Langer et al.. The significant differences between their studies and ours, include the fact that they studied mesh shrinkage using different mesh materials in an open retrorectus technique, in addition to using different imaging modalities for mesh surface calculation [10, 11]. In our study, the excellent inter-rater reliability among radiologists, together with the detailed MRI investigations, allowed us to correctly interpret mesh behavior in retrorectus robot-assisted minimal invasive ventral hernia repair. Demarcation of meshes via MRI imaging is a desirable noninvasive diagnostic tool which might guide the surgeon’s clinical practice in patients presenting with mesh-related symptoms, before directly applying revisional surgery. MRI depiction in our patient group, showed a remarkable streak of iron-loaded particles at the left lateral border of the mesh. We believe that this streak coincides with iron-loaded particles which were unleashed after tailoring the mesh to its appropriate measurement. Introducing the mesh into the retrorectus space, could have provoked iron-particles to stick to the opened ipsilateral posterior rectus sheath (Fig. 1).

### Generalizability

These study results are only applicable for robot-assisted minimal invasive retrorectus ventral hernia repair using an iron-oxide-loaded PVDF mesh and cannot be extrapolated for other mesh types or mesh positions which might influence mesh shrinkage to a significant extent. In addition, our clinical results might be representative of a specialized robotic abdominal wall surgeon and may not be translatable to the population of general surgeons. In addition, the short hospital stay is incentivized in our country, and may not represent the practice in other European countries where outpatient surgery may be financially disadvantageous.

## Supplementary Information

Below is the link to the electronic supplementary material.

Supplementary file1 (JPG 161 KB)

Supplementary file2 (JPG 672 KB)

Supplementary file3 (JPG 135 KB)

Supplementary file4 (DOCX 15 KB)

Supplementary file5 (DOCX 14 KB)
