# Supplementary figures and images for: Prospective cohort study on mesh shrinkage measured with MRI after robot-assisted minimal invasive retrorectus ventral hernia repair using an iron-oxide-loaded polyvinylidene fluoride mesh
Source: Surg Endosc. 2023 Feb 28;37(6):4604–12. doi: 10.1007/s00464-023-09938-3 (PMC10234924; doi:10.1007/s00464-023-09938-3)

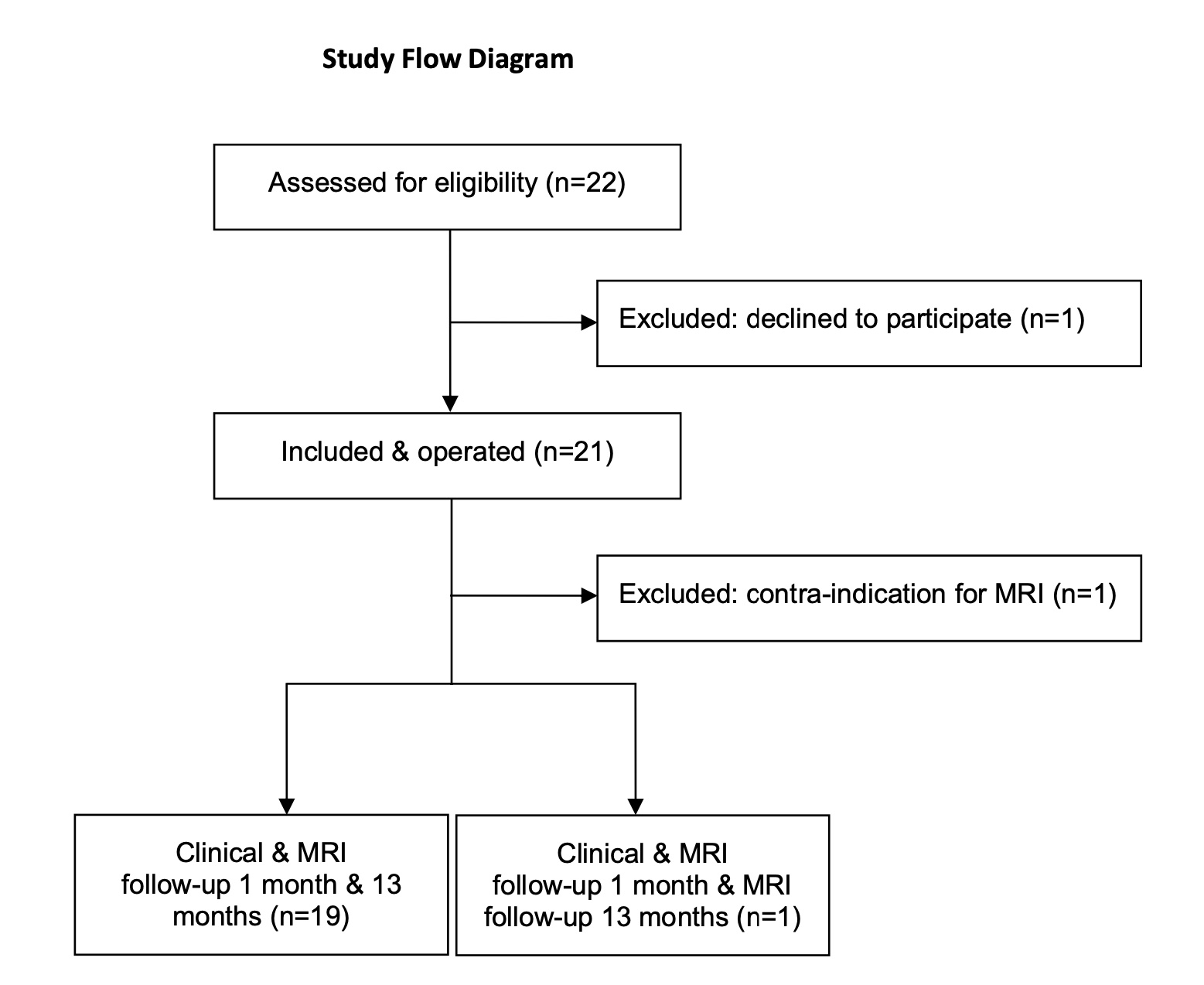

Supplement: Supplementary file 1 — Supplementary file1 (JPG 161 KB) [file 464_2023_9938_MOESM1_ESM.jpg]

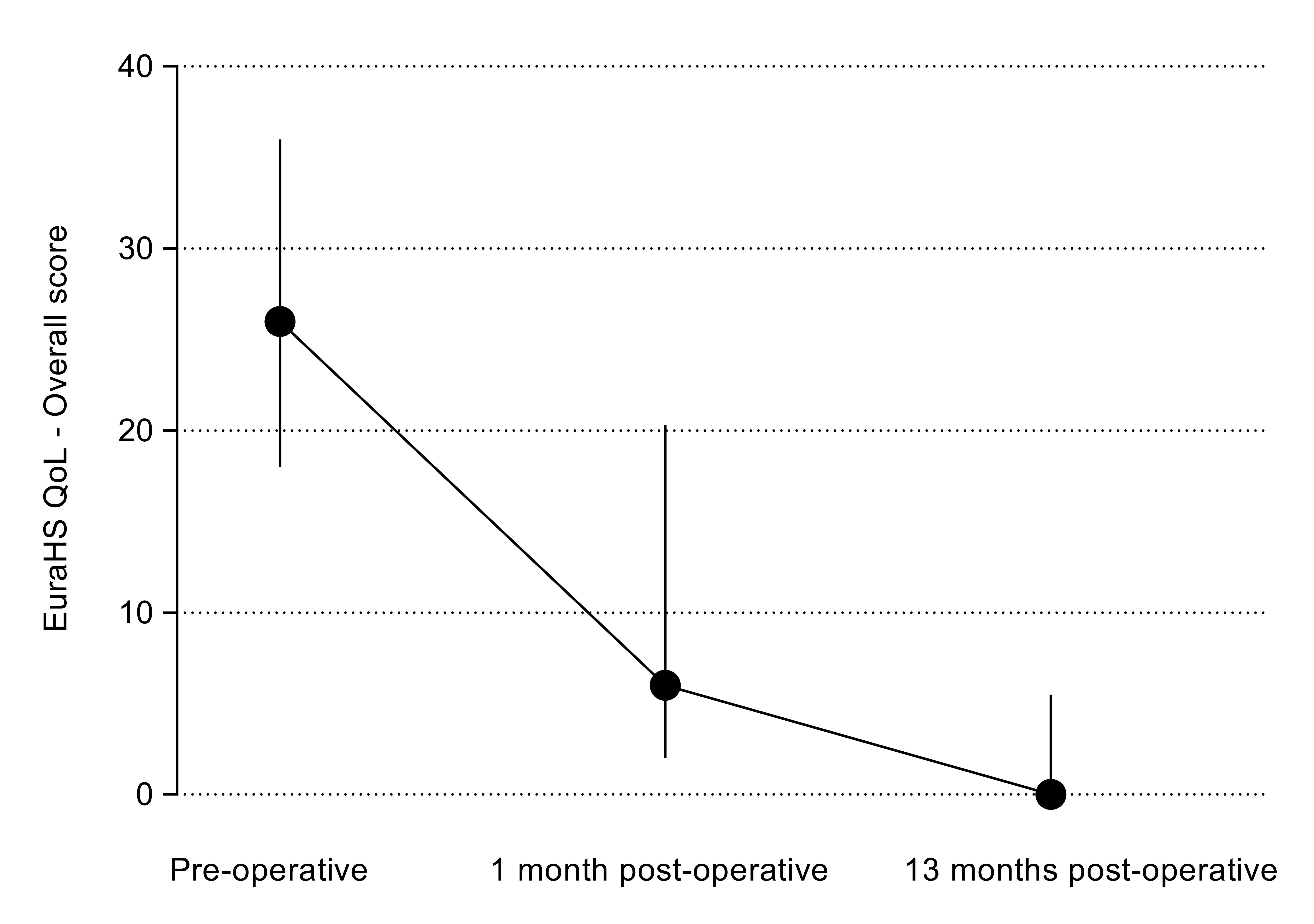

Supplement: Supplementary file 2 — Supplementary file2 (JPG 672 KB) [file 464_2023_9938_MOESM2_ESM.jpg]

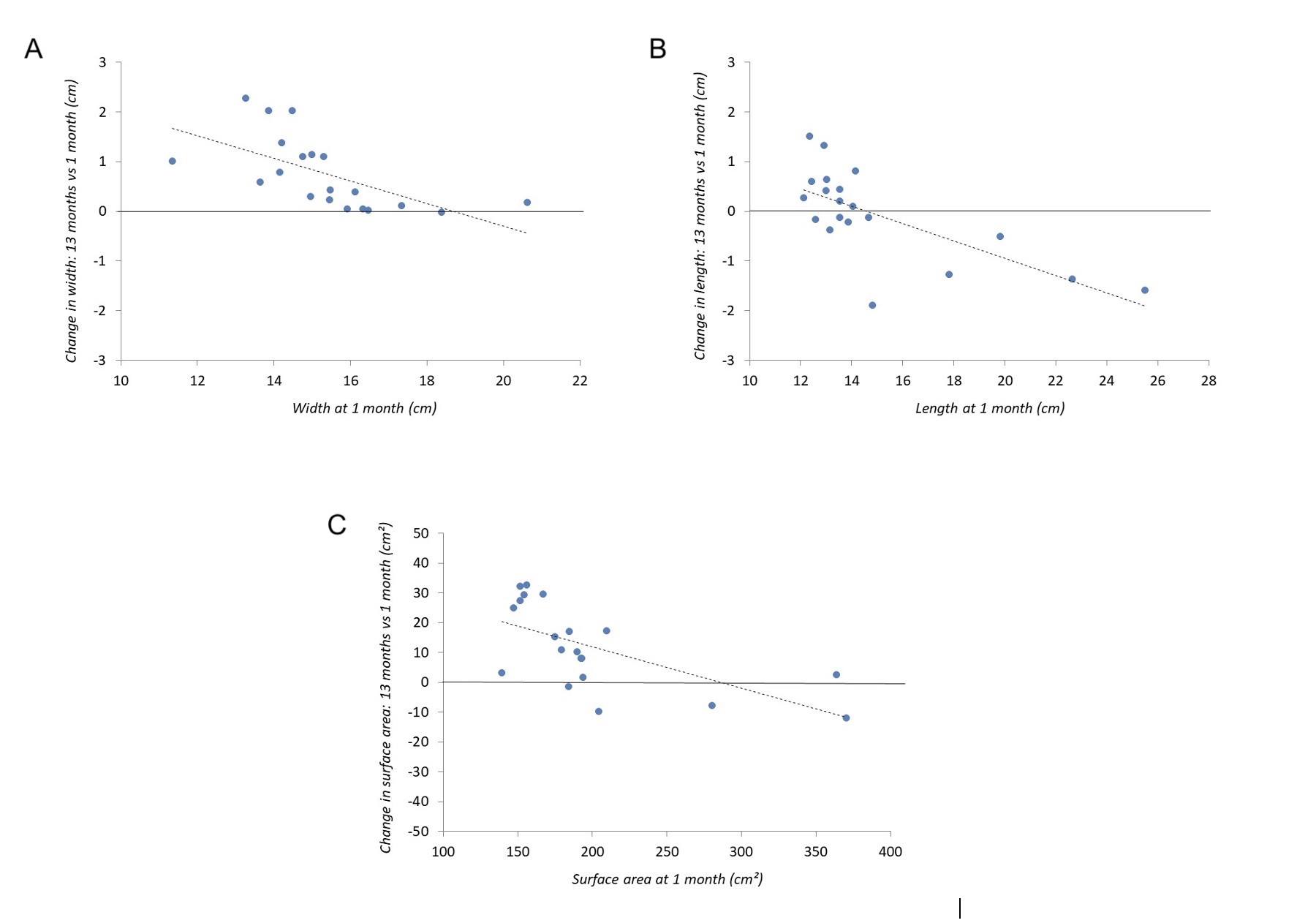

Supplement: Supplementary file 3 — Supplementary file3 (JPG 135 KB) [file 464_2023_9938_MOESM3_ESM.jpg]
