# Supplementary material for: Prospective cohort study on mesh shrinkage measured with MRI after robot-assisted minimal invasive retrorectus ventral hernia repair using an iron-oxide-loaded polyvinylidene fluoride mesh
Source: Surg Endosc. 2023 Feb 28;37(6):4604–12. doi: 10.1007/s00464-023-09938-3 (PMC10234924; doi:10.1007/s00464-023-09938-3)
Supplement: Supplementary file 4 — Supplementary file4 (DOCX 15 KB) [file 464_2023_9938_MOESM4_ESM.docx]

|  | EuraHS Quality of Life Scores | | |
| --- | --- | --- | --- |
|  | Pre-operatively | 1 month | 13 months |
|  |  |  |  |
| **Total** (/90) |  |  |  |
| Mean | 27.4 | 13.3 | 3.4 |
| Median (IR) | 26.0 (18.0-36.0) | 6.0 (2.0-20.3) | 0.0 (0.0-5.5) |
|  |  |  |  |
| **Pain** (/30) |  |  |  |
| Mean | 5.2 | 4.7 | 0.6 |
| Median (IR) | 3.0 (0.0-10.0) | 0.0 (0.0-7.0) | 0.0 (0.0-0.0) |
|  |  |  |  |
| **Restrictions** (/40) |  |  |  |
| Mean | 11.0 | 5.5 | 0.8 |
| Median (IR) | 11.0 (4.5-16.5) | 1.8 (1.0-8.5) | 0.0 (0.0-0.0) |
| **Cosmetic** (/20) |  |  |  |
| Mean | 11.2 | 3.2 | 2.0 |
| Median (IR) | 12.0 (9.0-14.0) | 2.0 (0.0-4.0) | 0.0 (0.0-4.5) |

Time effect (according to linear mixed modeling with unstructured covariance structure): Total score P<0.0001; Pain score P=0.017; Restrictions score P<0.0001; Cosmetic score P<0.0001
