# Supplementary material for: Prospective cohort study on mesh shrinkage measured with MRI after robot-assisted minimal invasive retrorectus ventral hernia repair using an iron-oxide-loaded polyvinylidene fluoride mesh
Source: Surg Endosc. 2023 Feb 28;37(6):4604–12. doi: 10.1007/s00464-023-09938-3 (PMC10234924; doi:10.1007/s00464-023-09938-3)
Supplement: Supplementary file 5 — Supplementary file5 (DOCX 14 KB) [file 464_2023_9938_MOESM5_ESM.docx]

|  | | 3DT1 MRI |  | IP FFE MRI |
| --- | --- | --- | --- | --- |
|  | Width (cm) | Length (cm) | Surface area (cm^2^) | Surface area (cm^2^) |
|  |  |  |  |  |
| Mean (SD) |  |  |  |  |
| Radiologist 1 | 15.7 (1.8) | 14.8 (3.4) | 204.0 (60.8) | 202.2 (59.6) |
| Radiologist 2 | 15.8 (1.9) | 15.1 (3.4) | 207.0 (60.2) | 204.2 (60.1) |
| Radiologist 3 | 15.7 (1.9) | 14.8 (3.3) | 204.8 (61.1) | 201.3 (60.9) |
| Significance | P=0.239 | P=0.0035 | P<0.001 | P<0.001 |
|  |  |  |  |  |
| Inter-rater reliability |  |  |  |  |
| ICC(2,3) (95% CI) | **0.95** (0.92-0.97) | **0.98** (0.97-0.99) | **0.99** (0.99-1.00) | **0.99** (0.99-1.00) |

*P-value obtained from repeated measures analysis of variance

Note 1: Results for radiologists 1 and 3 are not significantly different
Note 2: Measurements of radiologist 2 were significantly higher both at 1 month and 13 months
